# Drainage and Irrigation of the Antrum

**Published:** 1898-02

**Authors:** E. L. Townsend

**Affiliations:** Los Angeles, Cal.


ARTICLE V.

### DRAINAGE AND IRRIGATION OF THE ANTRUM.

BY E. L. TOWNSEND, D. D. S., LOS ANGELES, CAL.

Read before Pacific Coast Dental Congress, July 13, 1897.

Drainage, as applied to the antrum, means the opening and insertion of a tube into the antrum, allowing the contents to drain into the mouth, a practice that fails to accomplish the result desired; for, if the tube is short enough to allow the secretions to drain through the tube, granulations form about the end of the canal, producing more or less soreness and eventually closing the end of the opening. If the tube is long enough to prevent granulations forming over the end, it then prevents the very object it is intended to accomplish. Permanently fastening the tube to adjacent teeth is open to the objection that pus will seep between the tube and canal, thus becoming a source of irritation and infection, and establish a place that it is impossible to properly cleanse. And even if the drainage tube accomplished all that it is intended to do, it yet remains an undesirable method of treating these cases, on account of the disagreeableness of a constant dripping of pus into the oral cavity. A cleaner and more comfortable method is the complete plugging of the canal —the removal of the plug and a thorough irrigation of the antrum.

In making the opening into the antrum, a hole of sufficient size should be made to accommodate a plug made from No. 5 wire, standard gauge, having plugs and syringe nozzles drawn to the same gauge. In opening the antrum the hole should be made as direct as possible, and usually at a point occupied by the first molar. After the hole has been made, the plug is inserted, an impression taken and the plate made, which may carry the teeth or not, as circumstances dictate. During the interval between the taking of impression and insertion of the finished plate, a temporary plug should be worn, so that the hole shall not contract. This appliance soon becomes comfortable and is worn with no greater inconvenience than the ordinary denture, effectually preventing the contents of the antrum reaching the mouth, and upon its removal affording an opportunity for thoroughly cleansing the parts.

An ordinary fountain syringe, holding a quart or more, with a point bent to a right angle, is the most effective apparatus for irrigating the antrum. The syringe nozzle should be inserted into the opening, and the patient's head lowered to a point that will prevent any of the fluid running into the throat. A quart or more of slightly saline or alkaline solution is run through the antrum and out of the nose, thus completely washing the cavity and nasal passage. I have found it useless to try to cleanse the cavity by a spray or small amount of water. What it needs is a flood, something that will loosen the adherent, purulent mucous secretion and carry it away. The patient can attend to this, and irrigate as often as the conditions require, which ordinarily is from two to six times a day. It will be noticed that this treatment does not contemplate a speedy cure, but the preparations are made for a permanent opening and a lengthy treatment of the disease. In fact, I consider that the benefits arising from the opportunity to effectively cleanse the parts about the opening of the antrum into the nose compensate for the inconvenience and trouble. As most of the cases arise from a catarrhal condition, this gives an opportunity for treatment not offered by any other method, and so far as it has come under my observation it has been effective and has produced what is ordinarily termed a cure.—*Pacific Stomatological Gazette*, Sept., 1897.

